# Noninvasive Predictor for Premalignant and Cancerous Lesions in Endometrial Polyps Diagnosed by Ultrasound

**DOI:** 10.3389/fonc.2021.812033

**Published:** 2022-01-27

**Authors:** Jianying Xu, Xuan Rao, Weiguo Lu, Xing Xie, Xinyu Wang, Xiao Li

**Affiliations:** ^1^Department of Gynecologic Oncology, Women’s Hospital, School of Medicine Zhejiang University, Hangzhou, China; ^2^Cancer Center, Zhejiang University, Hangzhou, China; ^3^Center for Uterine Cancer Diagnosis & Therapy Research of Zhejiang Province, Hangzhou, China; ^4^Zhejiang Provincial Key Laboratory of Precision Diagnosis and Therapy for Major Gynecological Diseases, Women’s Hospital, Zhejiang University School of Medicine, Hangzhou, China

**Keywords:** endometrial polyps, hysteroscopy, malignancy, noninvasive predictor, ultrasound

## Abstract

**Background:**

There was no consensus for management of asymptomatic endometrial polyps (EPs) up to date.

**Objective:**

The aim of present study was to determine the risk factors of malignant lesions in EPs diagnosed by ultrasound and establish a noninvasive predictor to decrease unnecessary hysteroscopy for EPs.

**Study Design:**

We reviewed the records of all consecutive patients who underwent hysteroscopy for EPs in the Women’s Hospital School of Medicine Zhejiang University between January 1, 2001 and December 31, 2018. The patients with histological diagnoses of atypical hyperplasia or cancer were defined as malignancy, while the patients with histological diagnoses of benign lesions were randomly selected as benign group according to the ratio of 1:4 (malignancy:benign), matching by age and year of hospitalization. Logistic regression analysis was used to analyze the clinical parameters for predicting malignancy of EPs. A Chi-squared automatic interaction detection (CHAID) decision tree analysis was performed to find a noninvasive predictor. The sensitivity, specificity, and the receiver operating characteristic curve (ROC) were used for assess the efficacy of the noninvasive predictor. New diagnosed EPs patients received in 2019 were used for verifying the accuracy of the noninvasive predictor.

**Results:**

The age in 15,790 cases of benign lesions was significantly younger than that in 230 malignancy cases (41.97 ± 11.53 year vs 53.31 ± 11.61 years, p <0.001). AUB (OR 7.306, 95%CI 4.927–10.835), large EPs (OR 2.595, 95%CI 1.662–4.052), and blood flow signal in EPs (OR 2.690, 95%CI 1.872–3.866) were independent predictive factors of malignancy in all enrolled patients. A noninvasive predictor for malignancy of EPs was established, through combining with AUB, large polyps and blood flow signal. This predictor presented excellent sensitivity and NPV (91.3 and 95.8%), with acceptable specificity and AUC (0.801). Further validation in new diagnosed EPs also suggested excellent sensitivity and reasonable specificity (100 and 58.5%) of the predictor. Factors such as thickened endometrial thickness, menopause shorter than 10 years, hypertension, obesity and nulliparous were also validated as independent predictors of malignancy in different subgroup analysis.

**Conclusions:**

The noninvasive predictor combined with other risk factors from subgroup analysis would be reliable to distinguish the benign lesions from malignancy for EPs diagnosed by ultrasound.

## Introduction

Endometrial polyps (EPs) are defined as localized exophytic overgrowth occurring on the surface of the endometrium that may contain glands, fibrous tissue, and blood vessels in variable amounts (1, 2). With the widespread use of ultrasound in routine gynecological examination, the prevalence of EPs increased significantly, especially asymptomatic EPs (3). EPs may spontaneously regress in approximately 25% of cases, especially smaller polyps (<1.0 cm) (4). But the clinical manifestations of EPs were similar to early stage endometrial malignancy (3). The American Association of Gynecologic Laparoscopists (AAGL) estimated that the prevalence of cancerous and premalignant lesions of EPs range from 0 to 12.9%, depending on the subgroup (5). Thus, the fear of malignancy might inevitably entail overtreatment of EPs, which would bring unnecessary surgery related risks and lead to low cost-effectiveness ratio.

A number of studies have attempted to define the risk factors for cancerous and premalignant lesions of EPs diagnosed by ultrasound. Older age has been validated as a high-risk factor associated with malignancy. Previous reports suggested that the risk of malignancy increased over the age of 50, while younger age might be a protective factor (6, 7). Other studies also found that the incidence of malignancy increased 2.41–2.8 folds in women ≥60 years compared with those <60 years (1, 8). Abnormal uterine bleeding (AUB) is the most common symptom in EPs, with the incidence ranging from 23 to 75% (3, 9, 10). Previous studies also suggested that EPs patients with AUB presented an increased risk of malignancy (3, 11). For postmenopausal EPs, the women with AUB displayed an increased risk of malignancy ranging from 3.67 to 31.1 folds compared with asymptomatic women (1, 12), while for premenopausal EPs, AUB was not an independent risk factor for malignancy (1, 7, 13). Nevertheless, in order to relieve symptoms of AUB, surgical removal of EPs was recommended for symptomatic EPs patients in clinical practice, no matter whether for postmenopausal or premenopausal patients (5, 14).

The AAGL guidelines stated that expectant management was reasonable for small EPs in asymptomatic women (5). But the standard clinical management for asymptomatic EPs is still unclear to date (1, 7, 15). Moreover, studies about the relationship between obesity, diabetes mellitus (DM), hypertension, polycystic ovary syndrome (PCOS), menopause hormone replacement therapy (HRT), and malignancy of EPs also brought conflicting results (1, 9, 11, 13, 16, 17). So it is necessary to further comprehensively evaluate the risk factors related with malignant lesions in the EPs and create a more applicable strategy for clinical management of EPs. In the present study, we thoroughly analyzed the epidemiological and clinical parameters related to premalignant and cancerous lesions in EPs through various subgroup analyses, and firstly established a noninvasive comprehensive predictor which might be helpful to identify the malignant possibility of EPs patients. It is hoped to bring an excellent balance between missed diagnosis of the malignant lesions and unnecessary surgical complications and financial burden in EPs patients.

## Materials and Methods

We reviewed the records of all consecutive patients who underwent hysteroscopy for EPs in the Women’s Hospital School of Medicine Zhejiang University between January 1, 2001 and December 31, 2018. The enrolled patients should meet all the following inclusion criteria: EPs diagnosed by ultrasound preoperatively, EPs treated by hysteroscopy, histological diagnosis confirmed by Paraffin Section and used as gold standard for final definite diagnosis, clinical and demographic data were available. Patients with missing data were excluded. Clinical and demographic data were extracted from the medical records, namely, age at diagnosis, height and weight, gravidity and parity, menopausal status, AUB, the largest dimension and blood flow signal of the EPs in latest preoperative transvaginal sonography (TVS), past history such as hypertension, DM, breast cancer, Lynch syndrome, PCOS, family history of endometrial cancer and previous EPs confirmed by histology, the usage of HRT and tamoxifen, and the number and appearance of EPs observed by hysteroscopy, namely, size, shape, texture, surface vascular conditions and ulcerative conditions of the polyps. For postmenopausal women, the following variables were further collected, namely, age of menopause, the time after menopause (years), estrogen level, and endometrial thickness (ET) of single layer measured by TVS. The patients with histological diagnoses of atypical hyperplasia or cancer were defined as malignancy group, and further nominated as premalignant group and cancerous group respectively, while the patients with histological diagnoses of benign lesions were randomly selected as benign group according to the ratio of 1:4 (malignancy:benign), matching by age and year of hospitalization. Patients in the benign and malignancy groups were used as a training set for the modeling of the noninvasive predictor.

Furthermore, we retrieved all records of consecutive patients who underwent hysteroscopy for EPs in our hospital from January 1, 2019 to December 31, 2019. The inclusion and exclusion criteria were the same as mentioned above. The data collected were used as a validation set for verifying the noninvasive predictor. The present study was approved by the Medical Research Ethics Review Board of the Women’s Hospital School of Medicine Zhejiang University (IRB-20190016-R).

Ultrasound was carried out by the experienced sonographers with ultrasound systems equipped with a 5–9 MHz transvaginal probe. Body mass index (BMI) was calculated using weight divided by height squared (kg/m^2^). BMI ≥28 kg/m^2^ was defined as obesity. Women were considered menopause if they reported a period of amenorrhea of at least 12 months. Late menopause was defined as menopausal age >53 years. The appearance of EPs with crisp, prone to bleeding, irregular surfaces, ulcerative, and vascular abundance during hysteroscopy were considered as malignant signs (18).

Statistical analysis was carried out by Statistical Package for the Social Sciences (SPSS) software (version 24.0). Conditional univariate and multivariate logistic regression analyses were used to analyze the variable for predicting malignancy, and nonconditional univariate and multivariate logistic regression analysis were further used in subgroup analysis. Quantitative variables were presented as means ± standard deviation and compared by Student’s t-test. Qualitative variables were presented as number (percentage) and compared by Chi-square test. An alpha level <0.05 was considered statistically significant. The Chi-squared automatic interaction detection (CHAID) algorithm was used to perform decision tree analysis. The patients in the benign and malignancy groups were the training set. The groups created by the model of decision tree analysis were further divided into 3 subgroups according to the risk of malignancy: low-risk (≤5%), intermediate-risk (>5 to ≤20%), and high-risk (>20%). A noninvasive predictor was established based on decision tree analysis. The validation set was used to verify the accuracy of the noninvasive predictor. The sensitivity, specificity, positive predictive value (PPV), and negative predictive value (NPV) of the noninvasive predictor and direct judgment by hysteroscopy were calculated and compared for malignancy judgment. The area under the curve (AUC) with 95% CI was calculated using the receiver operating characteristic (ROC) curve.

## Results

### The Demographic and Clinical Data of Patients With EPs Diagnosed by Ultrasound

From January 1, 2001 to December 31, 2018, a total of 16,020 cases with clinical diagnosis of EPs were admitted for hysteroscopy in our hospital. Among them, 230 cases were histological diagnosed as malignancy, namely, 50 atypical hyperplasia and 180 cancers, while 920 patients with benign histological diagnoses were randomly selected as benign group, and 2 patients among them were further excluded due to a lack of sufficient information. The detailed histological results of all enrolled patients are listed in **Table 1**.

**Table 1:** Postoperative histological results of EPs diagnosed by ultrasound in training set.

Diagnosis	Number (%)
Premalignant and cancerous	230
Atypical hyperplasia	50 (21.74%)
Endometrial carcinoma	165 (71.74%)
Serous carcinoma	7 (3.04%)
Clear cell carcinoma	5 (2.17%)
Carcinosarcoma	3 (1.30%)
Benign	918
Endometrial polyps	880 (95.86%)
Simple hyperplasia	15 (1.63%)
Complex hyperplasia	6 (0.65%)
Uterine myoma	2 (0.22%)
Endometrial	15 (1.63%)

EPs, endometrial polyps.

As shown in **Table 2**, the age between malignancy and benign groups has no significantly difference due to age matched. But the age in 15,790 cases of benign lesions was significantly younger than that in malignancy (41.97 ± 11.53 years vs 53.31 ± 11.61 years, p <0.001). This result was consistent with previous reports (1, 7, 8). The incidence of AUB in benign group was significantly lower than that in malignancy (35.1% vs 82.2%, p <0.001). The dimension of EPs in benign group was smaller than malignancy (1.34 ± 0.71 cm vs 1.91 ± 1.00 cm, p <0.001), with 588 cases of large EPs (the largest dimension ≥1 cm) in benign group and 199 cases in malignancy. The ratio of blood flow signal detected by TVS in benign group was also significantly lower than that in malignancy (35.2% vs 64.3%, p <0.001).

**Table 2:** Demographic and clinical data in benign and malignancy group in training set.

Variables	Benign	Malignancy	Premalignant	Cancerous
Total	918	230	50	180
Age, y^a^	53.31 ± 11.59	53.31 ± 11.61	45.66 ± 11.95	55.44 ± 10.60
BMI, kg/m^2a^	23.38 ± 3.37	24.20 ± 3.65	23.95 ± 3.04	24.26 ± 3.80
Gravidity^a^	2.53 ± 1.41	2.58 ± 1.45	2.00 ± 1.26	2.74 ± 1.47
Parity^a^	1.54 ± 1.03	1.52 ± 1.05	0.98 ± 0.84	1.67 ± 1.06
Postmenopausal	574 (62.5%)	145 (63.0%)	15 (30.0%)	130 (72.2%)
Age of menopause, y^a,b^	50.37 ± 3.26	51.49 ± 3.50	52.53 ± 2.45	51.37 ± 3.59
The time after menopause, y^a,b^	10.00 ± 6.49	8.79 ± 7.01	6.13 ± 4.94	9.09 ± 7.16
Estrogen value ≥37pmol/l^b^	168 (29.3%)	40 (27.6%)	3 (20.0%)	37 (28.5%)
Late menopause	120 (13.1%)	49 (21.3%)	12 (24.0%)	37 (20.6%)
AUB	322 (35.1%)	189 (82.2%)	33 (66.0%)	156 (86.7%)
Largest dimension of EPs, cm^a^	1.34 ± 0.71	1.91 ± 1.00	1.59 ± 0.81	2.00 ± 1.03
Have blood flow signal	323 (35.2%)	148 (64.3%)	29 (58.0%)	119 (66.1%)
ET ≥0.2 cm^b^	103 (17.9%)	40 (27.6%)	7 (46.7%)	33 (25.4%)
Hypertension	258 (28.1%)	75 (32.6%)	15 (30.0%)	60 (33.3%)
DM	48 (5.2%)	12 (5.2%)	0 (0.0%)	12 (6.7%)
HRT	6 (0.7%)	1 (0.4%)	1 (2.0%)	0 (0.0%)
Breast cancer	25 (2.7%)	1 (0.4%)	0 (0.0%)	1 (0.6%)
Tamoxifen use	9 (1.0%)	0 (0.0%)	0 (0.0%)	0 (0.0%)
Lynch syndrome	0 (0.0%)	0 (0.0%)	0 (0.0%)	0 (0.0%)
PCOS	1 (0.1%)	3 (1.3%)	2 (4.0%)	1 (0.6%)
Family history of endometrial cancer	3 (0.3%)	0 (0.0%)	0 (0.0%)	0 (0.0%)
Previous EPs	72 (7.8%)	12 (5.2%)	6 (12.0%)	6 (3.3%)
Multiple polyps	424 (46.2%)	120 (52.2%)	35 (70.0%)	85 (47.2%)
Malignant sign by hysteroscopy	17 (1.9%)	98 (42.6%)	7 (14.0%)	91 (50.6%)

BMI, body mass index; AUB, abnormal uterine bleeding; EPs, endometrial polyps; ET, endometrial thickness; DM, diabetes mellitus; HRT, hormone replacement therapy; PCOS, polycystic ovary syndrome.

a It was expressed in terms of mean ± standard deviation.

b This variable was calculated only for postmenopausal patients.

Due to the clinical significance of AUB and menopause in EPs, the demographic and clinical data were further listed according 4 subgroups which included postmenopausal women with and without AUB, premenopausal women with and without AUB (**Appendix Table 1**). HRT, tamoxifen therapy, Lynch syndrome, PCOS and family history of endometrial cancer were excluded in further data analysis due to the very small sample size.

### Independent Risk Factors for Predicting Malignancy in Patients With EPs

As shown in **Table 3**, univariate analysis demonstrated that AUB, large EPs, blood flow signal in EPs and late menopause were significantly related with malignancy. Multivariate analysis further validated that AUB (OR 7.306, 95%CI 4.927–10.835), large EPs (OR 2.595, 95%CI 1.662–4.052), and blood flow signal in EPs (OR 2.690, 95%CI 1.872–3.866) were independent predictive factors of malignancy in all enrolled patients. When benign and premalignant patients were respectively compared with cancerous group, both postmenopause and AUB were validated as independent risk factors of cancer (**Appendix Tables 2.1**, **2.2**).

**Table 3:** Univariate and multivariate analysis of demographic and clinical data between benign and malignancy groups in training set.

	Variables	OR	95%CI	p-Value
Univariate	Obesity	1.483	0.960–2.291	0.076
	Gravidity (0 vs ≥1)	1.264	0.654–2.445	0.484
	Parity (0 vs ≥1)	1.475	0.821–2.653	0.193
	Postmenopausal	1.125	0.590–2.143	0.721
	Late menopause	1.996	1.328–2.999	0.001
	AUB	8.369	5.706–12.275	<0.001
	Large EPs	3.705	2.463–5.573	<0.001
	Have blood flow signal	3.675	2.659–5.078	<0.001
	Hypertension	1.286	0.918–1.802	0.144
	DM	1.000	0.520–1.925	1.000
	Breast cancer	0.147	0.020–1.104	0.062
	Previous EPs	0.639	0.338–1.207	0.168
	Multiple polyps	1.313	0.963–1.790	0.085
Multivariate	Late menopause	1.414	0.860–2.325	0.172
	AUB	7.306	4.927–10.835	<0.001
	Large EPs	2.595	1.662–4.052	<0.001
	Have blood flow signal	2.690	1.872–3.866	<0.001

CI, confidence interval; AUB, abnormal uterine bleeding; EPs, endometrial polyps; DM, diabetes mellitus.

Due to the menopause status and AUB were both important impact factors for clinical manage in EPs patients, subgroup analyses were conducted (**Tables 4**, **5** and **Appendix Tables 3, 4**). In addition to AUB, large EPs and blood flow signal, univariate and multivariate analyses suggested that ET ≥0.2 cm (OR 1.914, 95%CI 1.126–3.254) was an independent predictor of malignancy for postmenopausal patients, while hypertension (OR 5.891, 95%CI 1.392–24.937) was an independent predictor of malignancy for premenopausal patients (**Tables 4**, **5**). For postmenopausal patients without AUB, menopause <10 y (OR 3.205, 95%CI 1.024–10.000), blood flow signal (OR 2.865, 95%CI 1.143–7.177), and ET ≥0.2 cm (OR 3.624, 95%CI 1.397–9.397) were found to be independent predictors of malignancy. For premenopausal patients without AUB, nulliparous (OR 6.494, 95%CI 2.088–20.000), obesity (OR 8.867, 95%CI 1.512–52.008), large EPs (OR 4.180, 95%CI 1.283–13.616), and hypertension (OR 5.891, 95%CI 1.392–24.937) were found to be independent predictors of malignancy.

**Table 4:** Univariate and multivariate analysis of demographic and clinical data between benign and malignancy groups in postmenopausal patients in training set.

	Variables	OR	95%CI	p-Value
Univariate	Obesity	1.336	0.802–2.224	0.266
	Gravidity (0 vs ≥1)	0.655	0.145–2.959	0.582
	Parity (0 vs ≥1)	0.787	0.225–2.755	0.708
	The time after menopause <10 y	1.449	1.000–2.101	0.050
	Estrogen value ≥37 pmol/l	0.953	0.636–1.427	0.814
	Late menopause	2.104	1.370–3.232	0.001
	AUB	14.889	9.059–24.469	<0.001
	Large EPs	3.647	2.075–6.408	<0.001
	Have blood flow signal	4.270	2.918–6.249	<0.001
	ET ≥0.2 cm	1.742	1.142–2.657	0.010
	Hypertension	1.019	0.703–1.476	0.922
	DM	1.087	0.558–2.115	0.806
	Breast cancer	0.183	0.024–1.371	0.098
	Previous EPs	0.875	0.354–2.160	0.771
	Multiple polyps	1.228	0.840–1.794	0.288
multivariate	Late menopause	1.696	0.996–2.889	0.052
	AUB	13.630	8.140–22.822	<0.001
	Large EPs	2.944	1.539–5.630	0.001
	Have blood flow signal	3.264	2.058–5.176	<0.001
	ET ≥0.2 cm	1.914	1.126–3.254	0.017

CI, confidence interval; AUB, abnormal uterine bleeding; EPs, endometrial polyps; DM, diabetes mellitus; ET, endometrial thickness.

**Table 5:** Univariate and multivariate analysis of demographic and clinical data between benign and malignancy groups in premenopausal patients in training set.

	Variables	OR	95%CI	p-Value
Univariate	Obesity	2.130	0.880–5.157	0.148
	Gravidity (0 vs ≥1)	1.343	0.733–2.460	0.415
	Parity (0 vs ≥1)	1.486	0.867–2.551	0.190
	Late menopause	1.155	0.529–2.521	0.837
	AUB	3.781	2.195–6.516	<0.001
	Large EPs	3.706	2.068–6.644	<0.001
	Have blood flow signal	2.609	1.537–4.430	<0.001
	Hypertension	2.722	1.392–5.325	0.005
	DM	0.988	0.977–1.000	0.712
	Breast cancer	0.988	0.977–1.000	0.712
	Previous EPs	0.505	0.208–1.225	0.138
	Multiple polyps	1.568	0.897–2.743	0.143
multivariate	AUB	3.403	1.937–5.978	<0.001
	Large EPs	2.697	1.464–4.996	0.001
	Have blood flow signal	3.506	2.352–5.226	0.002
	Hypertension	2.744	1.297–5.804	0.008

CI, confidence interval; AUB, abnormal uterine bleeding; EPs, endometrial polyps; DM, diabetes mellitus.

### The Efficacy of Noninvasive Predictor in Predicting of Malignancy in EPs Diagnosed by TVS

**Figure 1:**
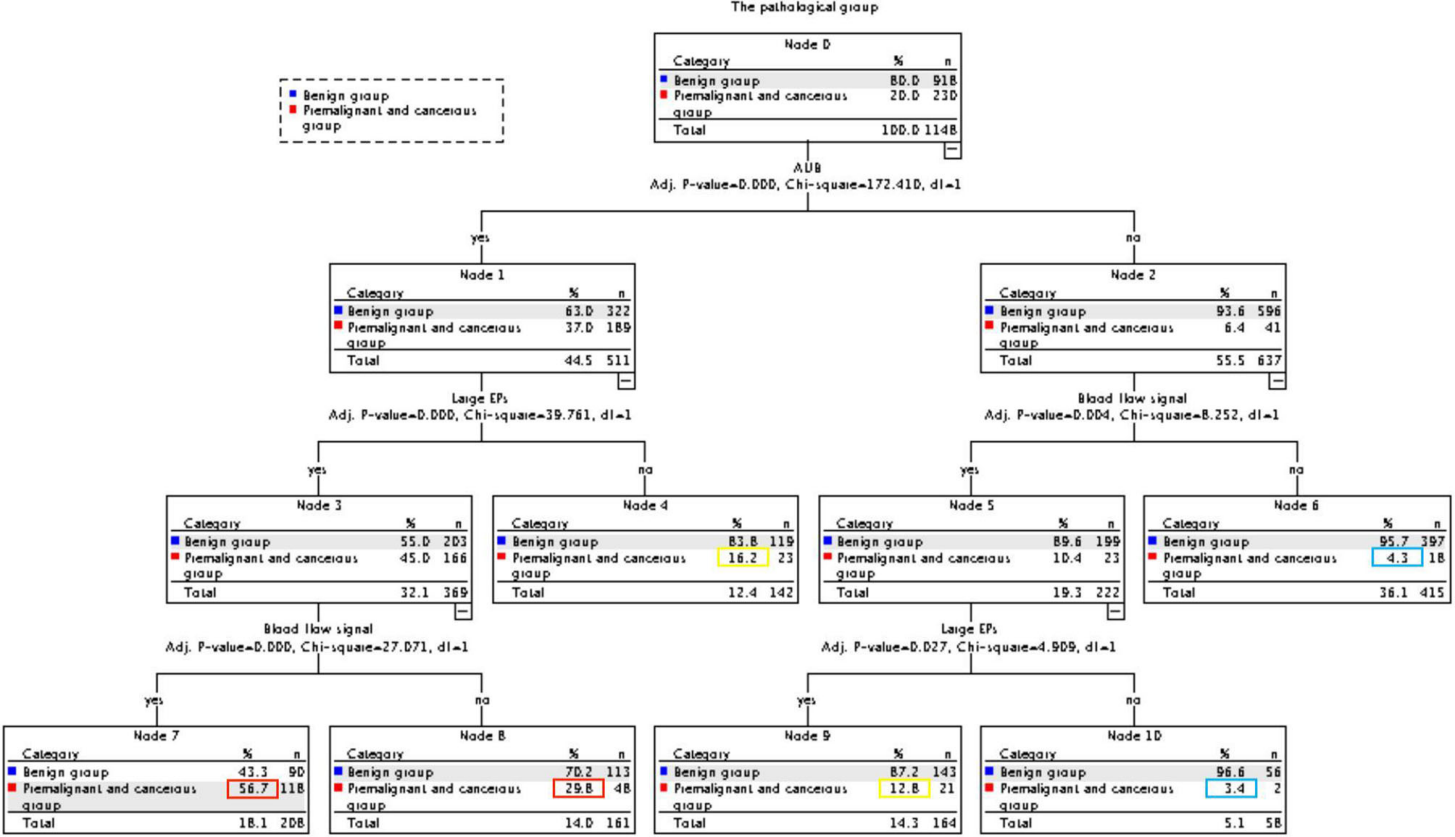
CHAID decision tree analysis on the risk of malignancy in EPs (Nodes 6 and 10 are classified as low-risk groups, 4 and 9 as intermediate-risk groups, 7 and 8 as a high-risk group).

**Figure 2:**
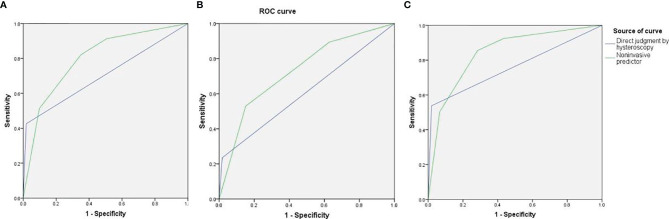
The area under the curve (AUC) of direct judgment by hysteroscopy and noninvasive predictor in total patients **(A)**, premenopausal patients **(B)**, and postmenopausal patients **(C)**.

As high risk factors for malignancy in EPs, AUB, large EPs and blood flow signal were further inputted into the model of decision tree analysis. The model created six groups which were further divided into low, intermediate, and high-risk subgroups for malignancy (**Figure 1**). The overall accuracy of decision tree analysis for correctly identifying malignant lesions in EPs was 82.4%. For internal validation, a misclassification risk of 18.3% ± 1.1% (standard error) was calculated using the 10-fold cross-validation method. Thus, EPs with AUB, or TVS showed large EPs and blood flow signal at same time were designated as noninvasive predictor, since they were created in the high-risk or intermediate-risk subgroups. When compared with the direct judgment by hysteroscopy, the predictor had significantly higher sensitivity (91.3% vs 42.6%, p <0.001) and NPV (95.8% vs 87.2%, p = 0.040) for malignancy. The AUC of the predictor was also higher than direct judgment by hysteroscopy (0.801 vs 0.704) (**Table 6** and **Figure 2**). Although the predictor showed lower PPV (31.1% vs 85.2%, p <0.001) and specificity (49.3% vs 98.1%, p <0.001), we thought it was reasonable as a primary screening test, especially in postmenopausal group (PPV = 35.0%, specificity = 56.6%). Furthermore, we also did the external validation of the decision tree model. We used the data of new diagnosed EPs in 2019 to verify the efficacy of the noninvasive predictor. A total of 1,863 EPs were diagnosed by ultrasound and underwent hysteroscopy in 2019. Among them, the malignancy group were 25 cases, namely, 12 atypical hyperplasia and 13 cancers. All the 25 patients had AUB or large EPs with blood flow signal. The average age in the validation set was consistent with that of 16,020 cases between January 1, 2001 and December 31, 2018 (40.61 ± 11.94 years vs 42.14 ± 11.94, p = 0.073). For external validation, the overall accuracy for identifying malignant lesions in EPs was 89.2%, while the misclassification risk was 10.8% ± 0.7% (standard error). Our predictor still presented excellent screening value in different subgroups, with specificity of 58.5% in total population and 71.6% in postmenopausal group (**Table 6**). These results suggested that the adoption of the predictor would lead to at least 40% reduction in unnecessary surgeries.

**Table 6:** The diagnostic value of direct judgment by hysteroscopy and noninvasive predictor.

Set	Method	Groups	Sensitivity (%)	Specificity (%)	PPV (%)	NPV (%)	AUC
Set 1	A	total	42.6	98.1	85.2	87.2	0.704
		premenopausal	23.5	98.3	76.9	83.9	0.609
		postmenopausal	53.8	98.1	87.6	89.4	0.759
	B	total	91.3	49.3	31.1	95.8	0.801
		premenopausal	89.4	37.2	26.0	93.4	0.731
		postmenopausal	92.4	56.6	35.0	96.7	0.843
Set 2	A	total	36.0	99.9	90.0	99.1	0.680
		premenopausal	21.4	99.9	75.0	99.3	0.607
		postmenopausal	54.5	100	100	98.5	0.773
	B	total	100	58.5	3.2	100	0.801
		premenopausal	100	55.6	2.0	100	0.774
		postmenopausal	100	71.6	10.5	100	0.899

Set 1, Training Set; Set 2, Validation Set; Method A, Direct judgment by hysteroscopy; Method B, Noninvasive predictor; PPV, positive predictive value; NPV, negative predictive value; AUC, area under the curve.

## Comment

As we know, hysteroscopy is recommended for EPs as both a diagnostic and therapeutic intervention (5). In the present study, all enrolled patients with EPs were subjected to hysteroscopy, which allowed us to assess the final diagnosis of the “EPs” accurately by histology results. We found that 4.14% of the patients with “EPs” in benign group were validated as other diseases by histology, namely, myomas, endometrium, etc., which suggested that TVS findings were not specific (5, 19, 20). More importantly, 1.44% of the patients with clinical diagnosis of EPs were finally diagnosed as malignancy. These results suggested that the indications of hysteroscopy should be developed carefully to balance missed diagnosis and overtreatment of EPs better. The challenge is how to choose the favorable opportunity to perform hysteroscopy.

Consistent with previous studies (1, 7, 8), our results suggested that age was a high risk factor for malignancy in “EPs” patients. The mean age of malignancy was about 11 years older than benign patients. In addition to age, other factors, namely, obesity and ultrasonic instruments can also worsen the diagnostic accuracy of transvaginal ultrasound (21, 22), so we matched the age and the year of hospitalization in training set in order to better evaluate other risk factors. Our results suggested that AUB, large EPs, and blood flow signal were independent predictive factors for malignancy, no matter in all enrolled EPs patients, postmenopausal group or premenopausal group. In addition, postmenopause and AUB were both found as independent risk factors for cancer, when compared with benign and premalignant patients respectively.

Among the risk factors for malignancy mentioned above, AUB was the most significant one (1, 3, 11, 12). The malignant risk of premenopausal patients with AUB was 3.403 folds higher than those without AUB in present study, which was discordant with previous reports (1, 7, 13). This could be attributed to different study populations and sample sizes. For example, the study by Uglietti enrolled 803 premenopausal patients with only 6 malignancies (7). Nevertheless, symptomatic EPs were suggested to be removed, because evidences reported that AUB would be resolving in 75–100% of cases (5, 14, 23). Reports from Ben-arie and Ferrazzi suggested that the largest diameter of EP larger than 1.5 and 1.8 cm were risk indicators for malignancy (24, 25), while our results suggested that the diameter of EP ≥1 cm was high risk factor (OR = 2.595), which was in accord with the study by Wang (2). There are limited data and even opposed opinion to support Doppler ultrasound aiding in the differentiation of hyperplasia and malignancy in polyps (5, 26–29), but our results suggested that blood flow signal detected by TVS increased the prevalence of malignancy (OR = 2.690).

Multiple studies reported that thickened ET in postmenopausal patients was related with malignancy in “EPs” patients (30–32), with the cutoff value ranging from 0.5 to 1.1 cm (double layer). Similarly, the present study found the prevalence of malignancy in postmenopausal patients with ET ≥0.2 cm (single layer) was higher (OR 1.914) than others, which supported that thickened ET should also be alerted. Furthermore, premenopausal patients with hypertension should be ruled out malignancy, due to the OR of hypertension was 5.891. Costa-Paiva and Baiocchi also reported that hypertension was a risk factor of malignant EPs (12, 15). Although the intrinsic mechanism was not clear, hypertension has been reported that it was closely associated with hormone-dependent tumors (33).

There was no consensus for management of asymptomatic EPs up to date (1, 7, 15, 25). Thus, we further analyzed the risk factors in patients without AUB. In addition to thickened ET and blood flow signal, menopause shorter than 10 years was validated as an independent predictor of malignancy for postmenopausal patients without AUB, while in addition to large EPs and hypertension, obesity and nulliparous were considered as high risk factors for premenopausal patients without AUB. These results would help gynecologists make clinical decision for “EPs” upon certain circumstance.

Based on our encouraging data, we established a noninvasive predictor for malignancy of EPs, through combining with AUB, large EPs, and blood flow signal. Compared with the direct judgment by hysteroscopy, this predictor presented excellent sensitivity and NPV, with reasonable specificity. The AUC of this predictor reached 0.801. Further validation using new data also suggested that noninvasive predictors were valuable as a screening indicator. Thus, the newly noninvasive predictor would undoubtedly contribute to rational clinical management for “EPs”.

To our best knowledge, this was the largest scale study to evaluate the risk factors related with malignancy of “EPs”, with more than 200 cases of malignancy (1, 7, 15, 25, 34–38). The large sample size permitted us to comprehensively assess the association between risk factors and malignancy through various subgroup analyses. Due to the retrospective nature of the present study, some potential risk factors might not be fully evaluated. Further prospective studies are warranted to verify the related predictor of malignancy in “EPs” patients.

## Conclusions

The present study validated that AUB, large EPs and blood flow signal were important independent high risk factors for malignancy of EPs. We firstly presented a noninvasive predictor, which included AUB, large EPs (≥1 cm), and blood flow signal shown by TVS. The predictor could effectively predict the malignancy in EPs diagnosed by TVS and help gynecologist accurately choose hysteroscopy for high risk patients. As the complement to the noninvasive predictor, thickened ET in postmenopausal patients, menopause shorter than 10 years in postmenopausal patients without AUB, hypertension in premenopausal patients, obesity and nulliparous in premenopausal patients without AUB should be a concern for the possibility of malignancy. With the predictor taken with the complements together, it would be reliable to distinguish the benign lesions from malignancy for EPs diagnosed by TVS.

## Data Availability Statement

The raw data supporting the conclusions of this article will be made available by the authors, without undue reservation.

## Author Contributions

JX: Data curation, formal analysis, software, and writing-original draft. XR: Data curation, methodology, writing-original draft. WL: Supervision, validation and writing-review and editing. XX: Supervision, and writing-review and editing. XW, XL: Conceptualization, supervision, and writing-review and editing. All authors contributed to the article and approved the submitted version.

## Conflict of Interest

The authors declare that the research was conducted in the absence of any commercial or financial relationships that could be construed as a potential conflict of interest.

## Publisher’s Note

All claims expressed in this article are solely those of the authors and do not necessarily represent those of their affiliated organizations, or those of the publisher, the editors and the reviewers. Any product that may be evaluated in this article, or claim that may be made by its manufacturer, is not guaranteed or endorsed by the publisher.
