# Supplementary material for: Noninvasive Predictor for Premalignant and Cancerous Lesions in Endometrial Polyps Diagnosed by Ultrasound
Source: Front Oncol. 2022 Jan 27;11:812033. doi: 10.3389/fonc.2021.812033 (PMC8828905; doi:10.3389/fonc.2021.812033)
Supplement: Supplementary file 1 [file Table_1.doc]

| Appendix Table 1 Demographic and clinical data in different subgroups | | | | | | | | |
| --- | --- | --- | --- | --- | --- | --- | --- | --- |
| Variables | Premenopausal | | | | Postmenopausal | | | |
|  | With AUB | | Without AUB | | With AUB | | Without AUB | |
|  | Benign | Malignancy | Benign | Malignancy | Benign | Malignancy | Benign | Malignancy |
| Total | 159 | 65 | 185 | 20 | 163 | 124 | 411 | 21 |
| Age, y ^a^ | 42.25±8.47 | 42.51±8.18 | 40.99±9.18 | 37.95±10.26 | 59.83±6.07 | 60.65±6.18 | 60.58±5.90 | 58.00±4.54 |
| BMI, kg/m^2 a^ | 22.78±3.15 | 23.38±2.63 | 22.06±2.99 | 24.14±5.27 | 24.76±3.99 | 24.65±3.79 | 23.62±3.09 | 24.05±3.64 |
| Gravidity ^a^ | 2.15±1.31 | 2.18±1.50 | 1.93±1.43 | 1.40±1.43 | 2.88±1.36 | 2.85±1.31 | 2.81±1.34 | 3.33±1.24 |
| Parity ^a^ | 1.28±0.83 | 1.06±0.73 | 0.96±0.76 | 0.80±1.24 | 2.01±1.13 | 1.85±1.04 | 1.72±1.02 | 1.67±0.97 |
| Age of menopause, y ^a,b^ |  |  |  |  | 50.45±3.13 | 51.44±3.55 | 50.33±3.32 | 51.76±3.24 |
| The time after menopause, y ^a,b^ |  |  |  |  | 9.37±6.75 | 9.22±7.34 | 10.25±6.37 | 6.24±3.74 |
| Estrogen value ≥37pmol/l ^b^ |  |  |  |  | 67(41.1%) | 33(26.6%) | 101(24.6%) | 8(38.1%) |
| Late menopause | 15(9.4%) | 8(12.3%) | 17(9.2%) | 1(5.0%) | 24(14.7%) | 37(29.8%) | 64(15.6%) | 3(14.3%) |
| Largest dimension of EPs, cm ^a^ | 1.26±0.74 | 2.06±1.32 | 1.07±0.53 | 1.47±0.79 | 1.52±0.88 | 1.97±0.84 | 1.42±0.66 | 1.48±0.59 |
| Have blood flow signal | 82(51.6%) | 50(76.9%) | 98(53.0%) | 13(65.0%) | 42(25.8%) | 75(60.5%) | 101(24.6%) | 10(47.6%) |
| ET≥0.2cm^b^ |  |  |  |  | 42(25.8%) | 31(25.0%) | 61(14.8%) | 9(42.9%) |
| Hypertension | 16(10.1%) | 11(16.9%) | 11(5.9%) | 5(25.0%) | 79(48.5%) | 50(40.3%) | 152(37.0%) | 9(42.9%) |
| DM | 2(1.3%) | 0(0.0%) | 2(1.1%) | 0(0.0%) | 15(9.2%) | 10(8.1%) | 29(7.1%) | 2(9.5%) |
| Breast cancer | 1(0.6%) | 0(0.0%) | 3(1.6%) | 0(0.0%) | 4(2.5%) | 1(0.8%) | 17(4.1%) | 0(0.0%) |
| Previous EPs | 21(13.2%) | 6(9.2%) | 24(13.0%) | 0(0.0%) | 5(3.1%) | 5(4.0%) | 22(5.4%) | 1(4.8%) |
| Multiple polyps | 112(70.4%) | 51(78.5%) | 125(67.6%) | 20(100.0%) | 56(34.4%) | 48(38.7%) | 131(31.9%) | 6(28.6%) |
| Malignant sign by hysteroscopy | 2(1.2%) | 19(29.2%) | 4(2.1%) | 1(5.0%) | 3(1.8%) | 68(54.8%) | 8(1.9%) | 10(47.6%) |
| Abbreviation: BMI, body mass index; AUB, abnormal uterine bleeding; EPs, endometrial polyps; ET, endometrial thickness; DM, diabetes mellitus; HRT, hormone replacement therapy; PCOS, polycystic ovary syndrome.  ^a^ It was expressed in terms of mean ± standard deviation.  ^b^ This variable was calculated only for postmenopausal patients. | | | | | | | | |

| Appendix Table 2.1 Univariate and multivariate analysis of demographic and clinical data between benign and cancerous groups | | | | |
| --- | --- | --- | --- | --- |
|  | Variables | OR | 95%CI | p Value |
| Univariate | Obesity | 1.469 | 0.906-2.382 | 0.118 |
|  | Gravidity(0 vs ≥1) | 1.085 | 0.574-2.049 | 0.803 |
|  | Parity(0 vs ≥1) | 1.152 | 0.649-2.041 | 0.629 |
|  | Postmenopausal | 1.558 | 1.095-2.217 | 0.014 |
|  | Late menopause | 1.725 | 1.146-2.597 | 0.009 |
|  | AUB | 12.031 | 7.667-18.880 | <0.001 |
|  | Large EPs | 4.229 | 2.631-6.798 | <0.001 |
|  | Have blood flow signal | 3.594 | 2.565-5.034 | <0.001 |
|  | Hypertension | 1.279 | 0.909-1.800 | 0.158 |
|  | DM | 1.295 | 0.673-2.489 | 0.439 |
|  | Breast cancer | 0.200 | 0.027-1.482 | 0.115 |
|  | Previous EPs | 0.405 | 0.173-0.947 | 0.037 |
|  | Multiple polyps | 1.042 | 0.757-1.436 | 0.799 |
| multivariate | Postmenopausal | 2.789 | 1.827-4.258 | <0.001 |
|  | Late menopause | 1.418 | 0.861-2.335 | 0.170 |
|  | AUB | 13.280 | 8.288-21.277 | <0.001 |
|  | Large EPs | 2.871 | 1.712-4.817 | <0.001 |
|  | Have blood flow signal | 3.506 | 2.352-5.226 | <0.001 |
|  | Previous EPs | 0.641 | 0.252-1.633 | 0.351 |
| Abbreviation: CI, confidence interval; AUB, abnormal uterine bleeding; EPs, endometrial polyps; DM, diabetes mellitus. | | | | |

| Appendix Table 2.2 Univariate and multivariate analysis of demographic and clinical data between premalignant and cancerous groups | | | | |
| --- | --- | --- | --- | --- |
|  | Variables | OR | 95%CI | p Value |
| Univariate | Age (≥50 vs <50) | 4.182 | 2.169-8.063 | <0.001 |
|  | Obesity | 0.945 | 0.382-2.341 | 0.903 |
|  | Gravidity(0 vs ≥1) | 0.439 | 0.163-1.182 | 0.103 |
|  | Parity(0 vs ≥1) | 0.288 | 0.125-0.665 | 0.004 |
|  | Postmenopausal | 6.067 | 3.051-12.061 | <0.001 |
|  | Late menopause | 0.819 | 0.390-1.722 | 0.599 |
|  | AUB | 3.348 | 1.620-6.921 | 0.001 |
|  | Large EPs | 1.893 | 0.826-4.337 | 0.131 |
|  | Have blood flow signal | 1.413 | 0.744-2.681 | 0.291 |
|  | Hypertension | 1.167 | 0.591-2.302 | 0.657 |
|  | Previous EPs | 0.253 | 0.078-0.822 | 0.022 |
|  | Multiple polyps | 0.383 | 0.196-0.751 | 0.005 |
| multivariate | Age (<50 vs ≥50) | 0.752 | 0.238-2.374 | 0.627 |
|  | Parity(0 vs ≥1) | 0.837 | 0.297-2.356 | 0.736 |
|  | Postmenopausal | 5.787 | 2.840-11.794 | <0.001 |
|  | AUB | 3.363 | 1.517-7.456 | 0.003 |
|  | Previous EPs | 0.216 | 0.059-0.789 | 0.020 |
|  | Multiple polyps | 0.723 | 0.326-1.603 | 0.425 |
| Abbreviation: CI, confidence interval; AUB, abnormal uterine bleeding; EPs, endometrial polyps. | | | | |

| Appendix Table 3 Univariate and multivariate analysis of demographic and clinical data between benign and malignancy groups in postmenopausal patients without AUB | | | | |
| --- | --- | --- | --- | --- |
|  | Variables | OR | 95%CI | p Value |
| Univariate | Obesity | 1.004 | 0.225-4.472 | 0.996 |
|  | Gravidity(0 vs ≥1) | 1.025 | 1.009-1.041 | 0.999 |
|  | Parity(0 vs ≥1) | 1.030 | 1.013-1.047 | 0.999 |
|  | The time after menopause<10y | 4.444 | 1.468-13333 | 0.008 |
|  | Estrogen value ≥37pmol/l | 1.889 | 0.761-4.687 | 0.170 |
|  | Late menopause | 0.904 | 0.259-3.157 | 0.874 |
|  | Large EPs | 2.388 | 0.690-8.258 | 0.169 |
|  | Have blood flow signal | 2.790 | 1.151-6.763 | 0.023 |
|  | ET ≥0.2cm | 4.303 | 1.739-10.648 | 0.002 |
|  | Hypertension | 1.278 | 0.526-3.103 | 0.588 |
|  | DM | 1.387 | 0.308-6.246 | 0.670 |
|  | Breast cancer | 0.959 | 0.940-0.978 | 0.999 |
|  | Previous EPs | 0.884 | 0.113-6.894 | 0.906 |
|  | Multiple polyps | 0.855 | 0.324-2.254 | 0.751 |
| multivariate | The time after menopause<10y | 3.205 | 1.024-10.000 | 0.045 |
|  | Have blood flow signal | 2.865 | 1.143-7.177 | 0.025 |
|  | ET ≥0.2cm | 3.624 | 1.397-9.397 | 0.008 |
| Abbreviation: CI, confidence interval; AUB, abnormal uterine bleeding; EPs, endometrial polyps; ET, endometrial thickness; DM, diabetes mellitus. | | | | |

| Appendix Table 4 Univariate and multivariate analysis of demographic and clinical data between benign and malignancy groups in premenopausal patients without AUB | | | | |
| --- | --- | --- | --- | --- |
|  | Variables | OR | 95%CI | p Value |
| Univariate | Obesity | 9.000 | 2.196-36.890 | 0.002 |
|  | Gravidity(0 vs ≥1) | 2.016 | 0.753-5.405 | 0.163 |
|  | Parity(0 vs ≥1) | 3.215 | 1.256-8.197 | 0.015 |
|  | Late menopause | 0.520 | 0.066-4.129 | 0.536 |
|  | Large EPs | 3.033 | 1.059-8.687 | 0.039 |
|  | Have blood flow signal | 1.649 | 0.629-4.319 | 0.309 |
|  | Hypertension | 5.273 | 1.618-17.183 | 0.006 |
|  | DM | 0.000 | 0.000 | 0.999 |
|  | Breast cancer | 0.000 | 0.000 | 0.999 |
|  | Previous EPs | 0.000 | 0.000 | 0.998 |
|  | Multiple polyps | 1.440 | 0.500-4.148 | 0.499 |
| multivariate | Obesity | 8.867 | 1.512-52.008 | 0.016 |
|  | Parity(0 vs ≥1) | 6.494 | 2.088-20.000 | 0.001 |
|  | Large EPs | 4.180 | 1.283-13.616 | 0.018 |
|  | Hypertension | 5.891 | 1.392-24.937 | 0.016 |
| Abbreviation: CI, confidence interval; AUB, abnormal uterine bleeding; EPs, endometrial polyps; DM, diabetes mellitus. | | | | |
